# Preclinical students’ experiences in early clerkships after skills training partly offered in primary health care centers: a qualitative study from Indonesia

**DOI:** 10.1186/1472-6920-12-35

**Published:** 2012-05-28

**Authors:** Doni Widyandana, Gerard Majoor, Albert Scherpbier

**Affiliations:** 1 Skills Laboratory and Department of Medical Education, Faculty of Medicine, Gadjah Mada University, Farmako Street no 1, Sekip Utara, Jogjakarta, 55281, Indonesia; 2 Institute for Education, Faculty of Health, Medicine and Life Sciences, Maastricht University, Universiteitssingel 60, 6229ER, Maastricht, The Netherlands

**Keywords:** Clinical skills training, Early clinical experiences, Clerkships

## Abstract

**Background:**

Students may encounter difficulties when they have to apply clinical skills trained in their pre-clinical studies in clerkships. Early clinical exposure in the pre-clinical phase has been recommended to reduce these transition problems. The aim of this study is to explore differences in students' experiences during the first clerkships between students exclusively trained in a skills laboratory and peers for whom part of their skills training was substituted by early clinical experiences (ECE).

**Methods:**

Thirty pre-clinical students trained clinical skills exclusively in a skills laboratory; 30 peers received part of their skills training in PHC centers. Within half a year after commencing their clerkships all 60 students shared their experiences in focus group discussions (FGDs). Verbatim transcripts of FGDs were analyzed using Atlas-Ti software.

**Results:**

Clerkship students who had participated in ECE in PHC centers felt better prepared to perform their clinical skills during the first clerkships than peers who had only practiced in a skills laboratory. ECE in PHC centers impacted positively in particular on students’ confidence, clinical reasoning, and interpersonal communication.

**Conclusion:**

In the Indonesian setting ECE in PHC centers reduce difficulties commonly encountered by medical students in the first clerkships.

## Background

Medical institutions across the world have installed clinical skills laboratories to train students’ clinical skills [1]. Skills laboratory are used to train pre-clinical, clinical, and post graduate students, however in this study we will focus on skills training for pre-clinical students. In skills laboratories, pre-clinical students practice their clinical skills with peers, manikins and simulated patients. Skills laboratories provide a safe environment for practice and learning processes therein can be well structured [2].

However, recent studies have indicated that pre-clinical students entering their clerkships may encounter difficulties when they have to apply clinical skills learned, especially in patient contacts [3-5]. For example, students feel anxious when they have to perform diagnostic or therapeutic procedures with patients because they fear to harm patients [6].

To mitigate transition problems as described above, Dornan et al. [7] recommended inclusion of early clinical experiences (ECE) in the curriculum for pre-clinical students. ECE are supposed to improve preparation of pre-clinical students for their clinical rotations by immersing them in the reality of the clinical environment, by facilitating application of knowledge to clinical practice, and by improving students’ motivation for learning.

In principle ECE can be offered in any clinical setting, including facilities for primary, secondary and tertiary health care. With respect to primary health care (PHC) some studies have shown the relative advantage of PHC centers to provide opportunities for “hands on” practice by pre-clinical students, availability of general practitioners (GPs) to supervise the students, and with respect to the scope of PHC, which next to patient care also includes community health education [8,9]. We have shown the advantages of PHC for practical training of pre-clinical students, compared with secondary and tertiary hospital settings in the Indonesian context [10]. Furthermore, we demonstrated that pre-clinical students who were trained in PHC centers felt better prepared for entering their clerkships. [11]. For this study, the same students involved in the previous one were interviewed for this study after they had entered the clinical phase of their studies.

This study was undertaken to explore the effect of ECE in PHC on students’ performance in their first clinical clerkships. To this aim, we compared experiences of students exclusively trained in a skills laboratory and peers for whom part of their skills training was substituted by ECE in PHC centers.

## Materials and Methods

This study was conducted with 60 pre-clinical students who were randomly sampled based on student numbers from a full class of 197 fourth year students of the Faculty of Medicine at Gadjah Mada University (FM-GMU) in Jogjakarta, Indonesia. These students were in the last semester of their pre-clinical studies since the medical curriculum of FM-GMU has a pre-clinical phase of 3.5 years (and a clinical phase of 1.5 years).

All 60 students sampled attended the regular clinical skills training program conducted in the skills laboratory. In duos or trios, 30 of these students were offered in addition an 11-day clinical skills training program in PHC centers (PHC-trained: PT). In the PHC centers PT students were supervised by GP’s who had been trained to become aware of the standard procedures for clinical skills as taught in FM-GMU’s skills laboratory. For their training, GP’s were invited to the skills laboratory to be introduced to the skills training program for pre-clinical students, to attend demonstrations of some skills trainings to instruct them how to train students on certain clinical skills.

In Indonesia, each sub-district has at least one PHC center (called *Puskesmas*). For example in 2000 in Jogjakarta a sub-district encompassed on average 35,278 people [12]. Each center hosts one or some doctors and paramedics, the latter including nurses, midwives, and pharmacists who may be involved in the teaching of pre-clinical students. The characteristics and the availability of PHC centers in the proximity of any medical school in Indonesia have led us to elect PHC centers to arrange for ECE for pre-clinical students, particularly to prepare these students for their clinical rotations [13]. Eleven PHC centers around Yogyakarta city were involved in this study. Each centre hosted groups of five or six students for 11 days dispersed over a five-week period. PT students were assigned to provide health services, patient management, and health promotion in communities. The other 30 students received for the same time span additional clinical skills training in the skills laboratory, facilitated by senior students trained to act as skills laboratory assistants (not PHC-trained: NPT). Those senior students had received intensive training from staff to assure that training given by them would be equal to that provide by skills laboratory teachers [14,15]. In tha additional program NPT students practiced (“doctor-to-patient”) communication, physical examination, procedural, and clinical reasoning skills in the laboratory with manikins, their peers and simulated patients.

After conclusion of their pre-clinical program students commenced their clinical rotations in the teaching hospital. Six months after commencing their clinical clerkships 30 PT and 30 NPT students were randomly invited to participate in Focus Group Discussions (FGDs). By that time, each student had completed three or four clerkships (out of the total of 14 clinical departments). During those periods, the students have opportunity to interact with real patients in particular clinical departments in the hospital, such as internal medicine, ENT, surgery, etc. Those students were randomly assigned to three focus groups of ten NPT or PT students, respectively.

FGDs were facilitated by the first author and a research assistant. Discussions were structured by the following questions: 1) What is your opinion about pre-clinical training in the skills laboratory as preparation for your clerkships?; 2) What difficulties did you encounter in your first clerkships?; 3) What is your opinion about ECE in PHC centers?; and 4) What is your impression of the performance of students in the other group, compared with the performance of your own group?”. Each focus group convened twice and each session continued until no more new items emerged.

FGDs were audiotaped and transcribed verbatim by a research assistant. Transcripts were analyzed using ‘inductive content analysis’ by three persons: the first author, a senior clinical supervisor from the teaching hospital, and a research assistant. The first author briefed his co-analyzers about the objective of the study and how to perform open coding using Atlas-Ti version 6. Each coder worked independently to analyze the texts and assigned codes in accord with the main categories (i.e., the four questions asked) and tried to distinguish sub-categories therein [16,17]. To finalize the coding process, a meeting was held with the three coders to discuss their results until consensus was reached. A written summary of the outcomes was distributed among all FGD participants for checking its validity and to solicit comments. Two research assistants met with groups of students and sent e-mails to those not attending to solicit their comments on the summary of the outcomes of the FGDs [18]. The design of this study was approved by the ethical committee of FM-GMU.

## Results

All 60 students attended at least one of the FGD sessions. Each small group had two FGD sessions; the number of participants in each FGD sessions varied between six to twelve.

In the coding process 1,433 issues were identified in the 12 FGD transcripts. During the meeting of the coders consensus was reached that ten sub-categories could be identified, based on the issues most frequently emerging in each main category. In reporting on the outcomes of the FGDs the focus will be on issues mentioned eight times more frequently in either NPT or PT groups to condense presentation of the data in Table 1. Of course all issues mentioned were recorded and the most striking ones will be presented in the text.

**Table 1:** Issues referred to eight times more frequently in FGDs with NPT and PT students

**Major categories and sub-categories**	**Issues**	**Groups**	
		**NPT**	**PT**
**Pre-clinical training in skills laboratory**			
Strengths of training in skills laboratory	Preparing for interpersonal communication	12*	4
Weaknesses of training in skills laboratory	Differences between skills laboratory’s guidelines and procedures on site	15	23
Suggestions to improve training in skills laboratory	Having more topics for practice including communication skills, pediatric examination, local language, clerkship preparation, etc.	38	25
**Difficulties in first clerkships**			
Difficulties with communication	Difficulties related to interpersonal communication	12	0
Difficulties with physical examination	Different guidelines from various supervisors	21	9
	Difficulty to recognize a pathological condition in patients	14	2
Difficulties with diagnostic and therapeutic skills	Differences between manikins and patients	17	2
	Afraid of harming the patient	10	1
General difficulties	Difficulties during adaptation period; responsibilities, equipments, procedures, etc	28	8
	Insufficient supervision	24	14
	Difficulties in collaboration with other health workers, health authorities, etc.	10	2
	Low confidence in front of patients	15	3
	Confused in front of patients	11	2
	Nervous in front of patients	11	1
	NPT students need to observe first before performing a clinical skill in first clerkships	15	2
	Difficulties with clinical reasoning ability	14	6
**Students’ opinion on ECE for pre-clinical students**			
Advantages	Having clinical experiences earlier	0	50
	Being aware of differences between skills laboratory and hospital reality	9	28
	Learning to cooperate with other health workers	5	18
	Practicing interpersonal communication	0	24
	Improving confidence	16	26
	Improving clinical reasoning	4	20
	Providing opportunities to practice clinical skills	2	21
	Motivating to practice clinical skills	5	18
	Learning that PHC is a comfortable environment for practice	0	10
Students’ suggestions for ECE	Effectiveness depends on supervisor’s support	16	28
	Hospital attachments are needed to observe advanced clinical procedures	13	2
**Students’ comments on comparing PT and NPT groups**			
PT students feel better prepared than NPT students in first clerkships	PT students demonstrate to be better prepared in their first clerkships	10	34

*Cumulative frequency by which a similar issue was mentioned in FGDs.

In the checking process with FGD participants only minor comments were made with respect to weaknesses of training in the skills laboratory and expectations to practice in PHC centers during the pre-clinical studies. Those comments have been integrated in the final outcome of the FGDs.

### Pre-clinical training in skills laboratory

NPT students more often made positive remarks about their preparation for interpersonal communication skills and had more suggestions for improvement of the skills training program than PT students. On the other hand, PT students more frequently recognized differences between standards for skills as taught in skills laboratory and the way those skills were performed in clinical practice. Both NPT and PT students frequently advised to include ECE in the pre-clinical curriculum.

“…the [ECE] program is good, because it improves students’ clinical performance [in early clerkship] … all [pre-clinical] students should have it from the beginning… (NPT/1/1)

### Difficulties met in first clerkships

In this main category large differences emerged in the number of difficulties mentioned by NPT students as compared with PT students. NPT students brought up 202 different problems whereas PT students mentioned only 52. Difficulties to perform clinical skills were mentioned 74 times by NPT students versus 14 times by PT students. Difficulties mentioned by NPT students related to communication skills, including difficulties with interpersonal communication (e.g. greeting, showing empathy), taking an anamnesis, and understanding the local dialect. NPT students had also encountered more difficulties than PT students with physical examination (e.g., in ophthalmology and neurology) and recognizing pathological signs in patients (e.g., wheezing sounds, liver enlargement, and cardiac murmurs). Both NPT and PT students frequently mentioned that different guidelines given by distinct clinical supervisors negatively impacted on their performance in physical examination. With therapeutic skills NPT students had suffered most from differences between manikins and patients, e.g. with insertion of intravenous lines, minor surgery, tracheal intubation, urethral catheterization, and assisting at a delivery. More often than PT students NPT students mentioned to be afraid to harm the patient.

“…when we insert an intravenous catheter or perform minor surgery on a manikin, they [the manikins] are not screaming, but pediatric patients are always screaming from the beginning (…) Performing wound suturing on a patient feels really different, [real] skin is much more stretchy…” (NPT/3/1)

Beyond difficulties with the application of clinical skills, NPT more frequently than PT students referred to difficulties in adapting to the clerkship environment, cooperation with other health workers, and with clinical reasoning. NPT students also more often than PT students mentioned low confidence, confusion, and anxiety in front of patients. NPT students shared that they first needed to watch a clinical procedure before performing it themselves.

“…Yes, indeed, because we [PT students] had earlier [clinical] experiences, we are confident and courageous if the supervisors give us opportunities to perform clinical procedures on patients, like inserting an intravenous infusion, venapuncture, minor surgery, delivering a baby, etc. … It is different with our [NPT] colleagues who prefer to observe prior to performing…” (PT/3/1)

Both NPT and PT students frequently stated that insufficient supervision had been a problem in their first clerkships.

### Students’ opinions on ECE for pre-clinical students

Based on their personal experiences, PT students commented extensively on their attachment in PHC centers. Based on their observations also students in NPT groups shared opinions on ECE. The main advantages of ECE identified by many PT students was the opportunity to practice clinical skills in a real health care setting. ECE made PT students aware of differences between the skills laboratory and a clinical environment. ECE had positively impacted on PT students’ motivation for learning, on their confidence, clinical reasoning, and interpersonal communication. More than NPT students, PT students were given opportunities by their clerkship supervisors to perform clinical skills.

“…in the ENT department [when students sought permission to perform a clinical skill] the supervisor would always ask: “Did you ever perform this skill [on a patient]?” (…) On the first day [of that clerkship] we [NPT students] observed our [PT] peers [performing that skill] and practiced with them. Then in the second day the supervisor would also allow us [NPT students] to perform that skill…” (NPT/1/2)

Both NPT and PT students recognized that the effectiveness of ECE depends on the quality of supervision in the PHC centers. In particular NPT students were worried that the range of clinical procedures which could be practiced in PHC centers would be too limited.

**“…opportunities to observe various clinical procedures are more available in the Sardjito [teaching] Hospital than in *Puskesmas *[PHC centers]…” (PT/2/1)**

In addition, NPT students recognized the advanced functioning of their PT peers in the first clerkships.

“…the way my peer [PT] student Tasya interacted with patients differed strongly from my [NPT student] performance … she looked ready [for practice] and knew what should she do with the patient… and I was not.” (NPT/1/1)

## Discussion

This study shows that students who participated in ECE in PHC centers had less difficulties than NPT students with the transition from the pre-clinical phase of their studies to the clerkships. In the setting of Indonesia PHC centers proved to be suitable to offer ECE to medical students.

PT students and NPT students spent the same time on clinical skills training but in different contexts. PT students had the opportunity to interact with real patients in PHC centres. We assume this context to be more challenging for them than for NPT students who expanded their training in the skills laboratory setting. Even though the NPT group cannot be regarded as a true ‘control group’, we feel the advantages of ECE as experienced by the PT students clearly demonstrate the advantage of inclusion of such activities in the pre-clinical medical curriculum.

ECE impacted in particular on students’ confidence, clinical reasoning abilities and interpersonal communication skills. With respect to confidence, PT as well as NPT students (based on experience and observation of peers, respectively) frequently felt ECE had or would boost their confidence. As far as the NPT students are concerned this may be explained by their anxiety and lack of confidence when in their first clerkships they were confronted with patients. Apparently PT students had passed this stage in the PHC centers, where possibly close supervision by GP’s had supported them in overcoming this barrier.

Expectations with respect to improving clinical reasoning through ECE were far more frequently voiced by NPT students than by PT students. Perhaps expectations were set too high in the NPT students who had not participated in ECE, or the NPT students were highly impressed by the progress PT students had made with respect to clinical reasoning during ECE, often demonstrated by their ability to appropriately perform an anamnesis and to arrive at a diagnosis.

Opinions of students with respect to interpersonal communication yielded the most convincing data in support of ECE. NPT students mentioned frequently to feel they had been adequately prepared for interpersonal communication by training in the skills laboratory, but apparently this perception was challenged in practice because PT students expressed this opinion less frequently than their NPT peers. On the other hand, when PT students had entered the clerkships ECE had apparently erased their difficulties with interpersonal communication, whereas understandably those difficulties persisted in NPT students. This explanation is corroborated by the high frequency by which PT students advocated ECE for practicing interpersonal communication skills.

The reduction of difficulties encountered by PT students in their first clerkships as compared to those encountered by their NPT peers resulted in frequent recommendation by students from both groups to include ECE in the pre-clinical curriculum. It is remarkable that NPT students did not mention the advantage of having clinical experiences in the pre-clinical curriculum, although in the first clerkships they had been impressed by the advanced functioning of their PT peers. Perhaps their failure to see this advantage reflects the doubt whether attachments to PHC centers can adequately prepare them to perform advanced clinical procedures.

The data gathered from the students confirm the shortcomings of clinical skills training in the skills laboratory to prepare students for the clinical phase of their studies as also observed by others [2,19,20]. Perhaps offering more sessions with simulated patients and patients manifesting certain pathological conditions (e.g. liver enlargement, heart murmurs), if possible situated in a hospital environment might compensate for some of the shortcomings of skills laboratory training as experienced by NPT students (and thus also by regular students) [21]. Furthermore, involving clinical supervisors in the hospital in trainings with (simulated) patients may assist in further adjustment of clinical procedures as taught in the skills laboratory and as performed in practice. On the other hand, ECE seems more efficient to improve preparation of pre-clinical students for their clinical rotations than considerable expansion of the skills laboratory training program. This may be particularly important in developing countries where budget restrictions call for the selection of the most cost-efficient solutions [11].

Insufficient supervision experienced in the clerkships by both NPT and PT students may have elicited their joint comment that also effectiveness of ECE in PHC relies on the quality of supervision. Also for this pilot experiment FM-GMU skills laboratory staff extensively informed supervisors-to-be in PHC centers about the pre-clinical students’ level of competency and their derived learning needs, and about standards for execution clinical procedures as taught in the skills laboratory [22,23]. Unfortunately in many developing countries (including Indonesia), PHC centers may be severely understaffed [11,24]. Therefore, in the selection of PHC centers for ECE availability of adequate numbers of supervising staff must be carefully considered, because medical students need close supervision particularly in their first encounters with patients [25].

In the Indonesian setting PHC centers proved to be suitable to offer ECE for pre-clinical students. Although more advanced equipment and procedures may not avail in PHC centers, this setting was apparently ‘clinical’ enough to satisfactorily prepare pre-clinical students for functioning in the hospital setting. Training in PHC centers also fits in with the policy of the Indonesian government which requires that every medical graduate must serve for at least half a year in PHC. Furthermore, ECE in PHC centers may motivate some students to opt for a future career in PHC [7].

A limitation of this study is that the assessment of the effectiveness of ECE in PHC centers was only based on student opinions. By involving students representing two different groups (i.e., PT and NPT) and by comparing their mutual opinions we aimed to improve the validity of these subjective data. Furthermore, we have taken the paucity of significant comments by FGD participants on the summary of the FGD sessions as support for the validity of the analyzing process as applied.

In a future study we intend to involve objective assessment tools to compare the clinical performance of NPT and PT students during their first clerkships. A suitable approach in this respect could be assessment by direct observation using the Mini-Clinical Evaluation Exercise (Mini-CEX) [25].

## Conclusion

In the setting of a developing country, an 11-day ECE program in PHC centers was demonstrated to significantly reduce difficulties as commonly encountered by pre-clinical students in their first clinical clerkships.

## Competing interests

The authors declare that they have no competing interests.

## Authors’ contributions

DW contributed to the conceptual and practical design of the study, collected data, analyzed and interpreted the data, and wrote the first draft of the manuscript. GM and AS contributed to the conceptual design of the study and reviewed and edited the manuscript. All authors approved the final version of the manuscript.

## Author's Information

DW is a staff member in the Skills Laboratory and Department of Medical Education, Faculty of Medicine, Gadjah Mada University, Jogjakarta, Indonesia. He is a PhD student in Maastricht University, The Netherlands.

GM is an Associate Professor based in the Institute for Education, Faculty of Health, Medicine and Life Sciences, Maastricht University, Maastricht, The Netherlands.

AS is Professor and Dean of the Faculty of Health, Medicine and Life Sciences, Maastricht University, Maastricht, The Netherlands.

## Pre-publication history

The pre-publication history for this paper can be accessed here:

http://www.biomedcentral.com/1472-6920/12/35/prepub
